# Clostridial Myonecrosis: A Comprehensive Review of Toxin Pathophysiology and Management Strategies

**DOI:** 10.3390/microorganisms12071464

**Published:** 2024-07-18

**Authors:** Hussain Hussain, Aya Fadel, Efrain Garcia, Robert J. Hernandez, Zahraa F. Saadoon, Lamia Naseer, Ekaterina Casmartino, Mohammad Hamad, Taylor Schnepp, Rehan Sarfraz, Sohair Angly, Arumugam R. Jayakumar

**Affiliations:** 1 Department of Internal Medicine, Kendall Hospital-HCA Florida Healthcare, Miami, FL 33136, USA; roberthernandezmd@gmail.com; 2 Department of Internal Medicine and Infectious Disease, Larkin Community Hospital, Miami, FL 33143, USA; egarcia@mybayshoremedical.com (E.G.); zahraa.fadhil0178@gmail.com (Z.F.S.); lamianaseer9@gmail.com (L.N.); ekaterinacfgos@hotmail.com (E.C.); mhamad1@sgu.edu (M.H.); taylor.schnepp916@gmail.com (T.S.); resarfraz@gmail.com (R.S.); sangly2@larkinhospital.com (S.A.); 3 Department of Internal Medicine, Ocean University Medical Center—Hackensack Meridian Health, Brick, NJ 08724, USA; ayafadel167@yahoo.com; 4 Department of Obstetrics, Gynecology and Reproductive Sciences, University of Miami Miller School of Medicine, Miami, FL 33136, USA

**Keywords:** gas gangrene, clostridium, diabetes, blood glucose, infection, fever, renal failure

## Abstract

Clostridial myonecrosis, commonly known as gas gangrene (GG), is a rapidly progressing and potentially fatal bacterial infection that primarily affects muscle and soft tissue. In the United States, the incidence of GG is roughly 1000 cases per year, while, in developing countries, the incidence is higher. This condition is most often caused by *Clostridium perfringens*, a Gram-positive, spore-forming anaerobic bacterium widely distributed in the environment, although other Clostridium species have also been reported to cause GG. The CP genome contains over 200 transport-related genes, including ABC transporters, which facilitate the uptake of sugars, amino acids, nucleotides, and ions from the host environment. There are two main subtypes of GG: traumatic GG, resulting from injuries that introduce Clostridium spores into deep tissue, where anaerobic conditions allow for bacterial growth and toxin production, and spontaneous GG, which is rarer and often occurs in immunocompromised patients. Clostridium species produce various toxins (e.g., alpha, theta, beta) that induce specific downstream signaling changes in cellular pathways, causing apoptosis or severe, fatal immunological conditions. For example, the *Clostridium perfringens* alpha toxin (CPA) targets the host cell’s plasma membrane, hydrolyzing sphingomyelin and phosphatidylcholine, which triggers necrosis and apoptosis. The clinical manifestations of clostridial myonecrosis vary. Some patients experience the sudden onset of severe pain, swelling, and muscle tenderness, with the infection progressing rapidly to widespread tissue necrosis, systemic toxicity, and, if untreated, death. Other patients present with discharge, pain, and features of cellulitis. The diagnosis of GG primarily involves clinical evaluation, imaging studies such as X-rays, computer tomography (CT) scans, and culture. The treatment of GG involves surgical exploration, broad-spectrum antibiotics, antitoxin, and hyperbaric oxygen therapy, which is considered an adjunctive treatment to inhibit anaerobic bacterial growth and enhance the antibiotic efficacy. Early recognition and prompt, comprehensive treatment are critical to improving the outcomes for patients affected by this severe and life-threatening condition.

## 1. Introduction

### 1.1. Background

*Clostridium perfringens* (CP), a bacterium discovered by William Welch in 1891, is a formidable pathogen implicated in a spectrum of diseases, ranging from mild food poisoning to life-threatening conditions such as gas gangrene (GG) [1,2]. This organism was initially called Bacillus aerogenes capsulatus [3]. It was subsequently renamed Bacillus perfringens and then *Clostridium welchii* [2,3,4]. It is currently known as *Clostridium perfringens*, which is a spore-forming Gram-positive anaerobic bacillus [4]. The incidence of CP in the United States is around 1000–3000 cases per year, with a mortality rate ranging from 30% in non-immunocompromised to 67% in immunocompromised patients [1,3,5]. Therefore, understanding the different types of GG and their underlying etiology is crucial for accurate diagnosis and optimal management. CP presents in two main forms: traumatic and spontaneous [1]. Traumatic GG is the most common form and typically arises following traumatic injuries that introduce CP spores into deep tissue [1,3,6]. These injuries may result from trauma such as crush injuries, compound fractures, or penetrating wounds, including those sustained during severe accidents like motor vehicle collisions or industrial accidents [1,7]. Spontaneous or hematogenous GG, caused by *Clostridium septicum* (CS) or *Clostridium novyi* (CN), occurs without evident external trauma and is often associated with underlying medical conditions or predisposing factors that facilitate bacterial dissemination, such as immunocompromised states, malignancy, and vascular insufficiency [1,3,7,8].

### 1.2. Ecology

CP, a versatile bacterium, occupies diverse ecological niches, reflecting its adaptive capabilities [6,9,10]. It is commonly found in soil and sediment, existing in a spore form that ensures survival during periods of environmental stress [10,11]. Water sources such as lakes, rivers, and sewage can also harbor CP, especially in areas where human or animal waste contributes to contamination [12]. Additionally, it resides as part of the normal flora in the gastrointestinal tracts of humans and animals, where it aids in digestion, without causing harm under normal conditions [13].

The bacterium’s ability to form highly resistant spores is crucial to its environmental resilience [14,15]. These spores persist in soil and water, serving as reservoirs that can lead to infections under favorable conditions [15]. Agricultural practices and human activities that disturb soil or contaminate water can increase the spread of spores, heightening the risk of infection through ingestion or contact [15]. The transmission of CP typically occurs through the fecal–oral route, where the ingestion of contaminated food or water allows spores to survive acidic stomach conditions and germinate in the anaerobic environment of the intestines [1,2,14]. To mitigate these risks, effective public health measures focus on proper sanitation, hygiene practices, and food safety protocols.

### 1.3. Pathogenicity

The 632 strains of *C. perfringens* were classified into seven types (A–G) based on the latest updated classification. In brief, *C. perfringens* possesses a single circular chromosome ranging in size from 2.9 to 4.1 Mb [13,16,17]. It encodes approximately 2600 to 3800 predicted genes [18]. CP requires various essential nutrients and amino acids for proliferation, as revealed by genome sequencing, which indicates a lack of genes for the biosynthesis of many amino acids and the tricarboxylic acid cycle [18,19]. However, the genome encodes degradative enzymes, such as sialidases, and a complete set of enzymes for fermentation and glycolytic pathways, enabling the bacterium to utilize complex host carbohydrates by breaking them down into simple sugars [18,19].

The CP genome also contains more than 200 transport-related genes, including ABC transporters, which facilitate the uptake of sugars, amino acids, nucleotides, and ions from the host environment [18]. These transporters help the bacterium to compensate for its inability to synthesize essential nutrients by acquiring them from host tissue. Furthermore, the genome features a large number of rRNA operons and tRNAs, allowing for the rapid production of secreted enzymes and toxins [19]. This capacity for fast growth is a critical aspect of the organism’s virulence and its ability to outcompete other bacteria in the decomposition of dead tissue.

Plasmids are pivotal in the pathogenicity of CP, particularly in diseases originating in the intestines [18,19]. These extrachromosomal DNA elements carry genes that enhance the bacterium’s virulence and adaptability [18]. CP plasmids are categorized into three primary families based on the genes responsible for initiating plasmid DNA replication: pCW3-like, pCP13-like, and pIP404-like plasmids [5,18,19].

The plasmids in the pCW3-like family are conjugative, meaning that they can transfer from one bacterial cell to another through direct contact. This ability facilitates the spread of virulence factors among CP populations [19]. The pCW3-like plasmids often carry genes encoding toxins, antibiotic resistance, and other factors that enhance bacterial survival and virulence in the host environment [18,19].

Similar to the pCW3-like family, pCP13-like plasmids are also conjugative [20]. They play a crucial role in horizontal gene transfer, contributing to the genetic diversity and adaptability of CP [20]. These plasmids may harbor genes for enterotoxins, which are critical in food poisoning and other gastrointestinal diseases caused by CP [15]. The conjugative nature of these plasmids aids in the rapid dissemination of virulence genes within bacterial communities [19,20].

In contrast, pIP404-like plasmids are non-conjugative, meaning that they cannot transfer between bacterial cells through conjugation [20]. Despite this limitation, they still play a significant role in the pathogenicity of CP. These plasmids can carry genes that contribute to the bacterium’s ability to cause disease, such as those coding for toxins and other virulence factors. Their replication within a bacterial cell can enhance the overall virulence and survival capabilities of CP [5]. The presence of these plasmids in CP significantly enhances its ability to cause disease [20]. They encode a variety of virulence factors, including toxins, antibiotic resistance, and adaptation and survival mechanisms. Many plasmids carry genes for potent toxins, such as enterotoxins and beta toxins, which are responsible for the severe symptoms of food poisoning and GG [18,20]. Some plasmids harbor genes that confer resistance to antibiotics, complicating the treatment options and promoting the persistence of infections. Additionally, plasmids contribute to the bacterium’s ability to adapt to different environmental conditions, including those within the host, enhancing its survival and proliferation [20].

The *cpe* gene, encoding CP enterotoxin (CPE), is unique in that it can be found on either the chromosome or plasmids [18,21,22,23,24,25,26,27]. About 70% of type F human food poisoning isolates have their *cpe* genes located on the chromosome [22]. In these cases, the gene is often flanked by IS1470 sequences, suggesting that the chromosomal presence of the *cpe* gene may result from the integration of a *cpe*-carrying transposon [18,21]. The remaining 30% of type F food poisoning strains, nearly all type F non-food-borne human gastrointestinal disease strains, and *cpe*-positive type C, D, and E strains carry their *cpe* genes on large pCW3-like conjugative plasmids [18,22]. In type F strains, the *cpe* plasmids predominantly cluster into two sub-families: pCPF4969-like plasmids and pCPF5603-like plasmids [18,22]. The pCPF4969-like plasmids do not carry the cpb2 gene, while the pCPF5603-like plasmids do include the cpb2 gene [18,22,27].

Initially, CP spores play a crucial role in its pathogenesis [15]. These spores exhibit resistance to heat, cold, osmotic pressure, chemicals, and pH extremes, which facilitates CP’s survival in various ecosystems, particularly in type C and F strains [18]. A key factor in the spores’ resistance is the presence of α/β-type small acid-soluble proteins (SASPs), which bind to the spore’s DNA, shielding it from environmental stresses [15,23]. CP produces four primary SASPs, all contributing to spore resilience against heat, chemicals, and UV radiation [23].

CP enters tissue through multiple mechanisms, such as accidental traumatic injuries (e.g., compound fractures, penetrating war wounds, surgical wounds following procedures like bowel or biliary tract surgery, or septic abortion) [1,15,18]. Rarely, CP infection occurs with arterial insufficiency or after the parenteral injection of medications like aqueous epinephrine, subcutaneous insulin, or drugs such as methamphetamine and heroin [1,2]. CP has even complicated routine medical procedures like venipuncture or platelet infusions in patients with granulocytopenia [24].

In some cases, CP spreads via the bloodstream, establishing an infection without significant tissue injury, often associated with intestinal tract abnormalities such as colon cancer, diverticulitis, or bowel infarction [1,18,25]. Predisposing conditions include leukemia, neutropenia, and diabetes mellitus [25]. The bacterium likely enters through mucosal ulceration or perforation in the intestinal tract, sometimes leading to rapid multifocal muscle involvement or deep tissue injury [7,25]. The local tissue scattering of CP enhances the production of virulence factors.

**Figure 1:**
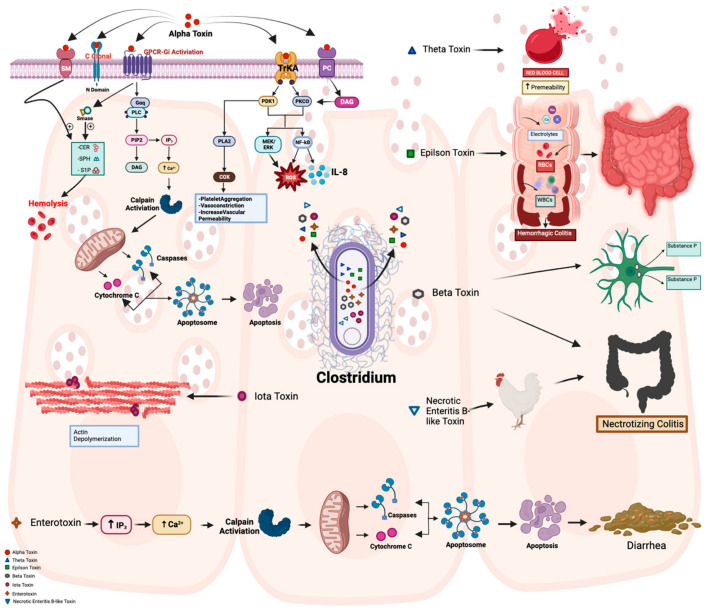
Clostridium virulence factors and their mechanisms of action. Alpha toxin targets host cell plasma membranes, hydrolyzing sphingomyelin and phosphatidylcholine, which activates phospholipases and sphingomyelinases, leading to calcium influx, calpain activation, and cell necrosis. Alpha toxin also interacts with Gi-GTP-BP and TrkA receptors and induces signaling cascades involving MAPK/ERK and NF-κB, resulting in ROS and IL-8 production and vascular effects. Theta toxin binds cholesterol, forming β-barrel pores, causing red blood cell lysis. Epsilon toxin creates heptameric pores, altering the ion balance and prompting caspase-independent cell death, leading to enterocolitis. Beta toxin affects neurons and colonic mucosa, inducing catecholamine release, arterial constriction, and TNF-α-mediated plasma extravasation, contributing to necrotic cell death and necrotizing enterocolitis. Iota toxin ADP-ribosylates actin, causing cell death. Enterotoxins disrupt intestinal mucosa tight junctions, form β-barrel pores, and trigger calcium influx, leading to calpain activation, necrosis, apoptosis, and diarrhea. Necrotic enteritis beta-like toxin in chickens forms heptameric pores, causing osmotic cell lysis. Mitogen-activated protein kinase/extracellular signal-regulated kinase, MAPK/ERK; nuclear factor kappa B, NF-kB; Gi-type GTP-binding protein, Gi-GTP-BP; reactive oxygen species, ROS; IL, interleukin; tumor necrosis factor, TNF; adenosine diphosphate, ADP.

The combination of toxin production (a minimal lethal dose in animals is 10^10^), enzymatic activity (e.g., proteases, hyaluronidase, collagenase, sialidases, endoglycosidases), and other virulence factors leads to extensive tissue necrosis [18,26,27]. Some toxins exert local tissue effects, while others cause systemic effects such as hemolysis, intravascular thrombosis, and cytokine release. This cascade can lead to shock, multi-organ failure, and ultimately death [26]. Various CP toxins, along with their mechanisms of action, are discussed below (Figure 1) [27,28,29,30,31,32,33,34,35,36,37,38,39,40,41,42,43,44,45,46,47,48,49,50,51,52].

### 1.4. Toxin Classification

Virtually all CP isolates produce alpha toxin (CPA), a zinc-containing phospholipase C enzyme consisting of 370 amino acids [18,53]. CPA targets the host cell’s plasma membrane, where it hydrolyzes sphingomyelin (SM) and phosphatidylcholine (PC), initiating a cascade of events [18,53,54]. CPA comprises distinct domains: the binding C-domain (C), catalytic N-domain (N), and ganglioside-binding loop domain (L) [18,53,54]. CPA interacts with the Gi-type GTP-binding protein (Gi-GTP-BP) on the plasma membrane, triggering the activation of endogenous phospholipases (PI-PLC) and sphingomyelinases (SMase) [18,53,54,55,56]. This leads to the production of diacylglycerol (DAG) and inositol trisphosphate (IP3), resulting in elevated calcium levels that activate cytoplasmic calpain, leading to necrosis through the release of mitochondrial cytochrome c and the formation of apoptosomes, ultimately resulting in apoptosis [18,55]. Simultaneously, SMase activity generates ceramide (CER), sphingosine (SPH), and sphingosine-1-phosphate (S1P), which induce the hemolysis of red blood cells [18,36,39,53,54,55]. CPA’s interaction with the TrkA receptor triggers the phosphorylation of 3-phosphoinositide-dependent protein kinase-1 (PDK1) and protein kinase C-theta (PKCθ) [18,36,39,53,54,55], activating the mitogen-activated protein kinase (MAPK)/MAPK kinase (MEK)/extracellular signal-regulated kinase (ERK) or MEK/ERK signaling cascade, as well as nuclear factor kappa B (NF-κB) [18,36,39,53,54,55]. NF-κB activation is associated with the formation of reactive oxygen species (ROS) and interleukin-8 (IL-8) [18,36,39,53,54,55]. Additionally, PDK1 phosphorylation activates phospholipase 2, leading to cyclooxygenase (COX) activation, which catalyzes the formation of prostaglandins, thromboxane, and leukotrienes [18,36,39,53,54,55]. These mediators facilitate vasoconstriction, enhance the vascular permeability, and promote platelet aggregation [57].

Theta toxin (perfringolysin O) is considered one of the pore-forming toxins, primarily targeting red blood cells and leading to coagulative necrosis [49,50,57]. Upon binding to cholesterol, the toxin diffuses through the plasma membrane bilayer, forming extensive β-barrel pore complexes [49,50]. These pores perforate the membrane, resulting in the release of intracellular content and ultimately causing the lysis of red blood cells [49,50]. Theta toxin has also been identified in Streptococcus, Listeria, and Bacillus [49,50].

Beta toxin is a pro-toxin composed of 336 amino acids. During secretion, it undergoes cleavage, removing a 27-amino-acid sequence [18,58,59,60,61,62,63,64]. The toxin acts as another pore-forming toxin by releasing substance P, which causes neurotoxicity and is involved in the pathogenesis of necrotizing colitis [18,58,59,60,61,62,63,64]. It exhibits dual affinity, impacting both neurons and the colonic mucosa [18]. Beta toxin acts as an agonist of the tachykinin NK1 receptor on autonomic nervous system neurons, prompting catecholamine release, arterial constriction, and increased blood pressure [18,58,59,60,61,62,63,64]. Additionally, beta toxin triggers substance P release, leading to neurogenic plasma extravasation [18,58,65,66]. Substance P, in turn, stimulates the release of tissue necrosis factor alpha (TNF-α), contributing to plasma extravasation [18,58,59,60,61,62,63,64]. Beta toxin’s role in intestinal endothelial cell necrosis involves cell signaling events that participate in calpain activation, ultimately leading to necrotic cell death and the development of necrotizing enterocolitis [18,58,59,60,61,62,63,64,66]. Recent studies have indicated that platelet endothelial cell adhesion molecule-1 (CD31 or PECAM-1) serves as a receptor for beta toxin on endothelial cells [18,65]. These studies demonstrate that the ectopic expression of CD31 in naturally resistant mouse epithelial cells allows them to bind beta toxin and form beta toxin oligomers, thereby rendering these cells sensitive to its toxicity [18,58,59,60,61,62,63,64]. Moreover, beta toxin is highly sensitive to trypsin and other intestinal proteases. To maintain its activity in vivo, the toxin requires trypsin inhibitors [18,65]. The affinity of beta toxin for other cell types is the subject of ongoing research [18].

The CP enterotoxin is a 35 kDa protein produced by all type F strains and certain type C, D, and E strains [18]. Unlike other toxins, enterotoxin does not exhibit significant amino acid sequence similarity but is classified structurally within the aerolysin β-pore-forming toxin family [67]. It consists of a C-terminal receptor-binding domain and an N-terminal cytotoxicity domain, which facilitates oligomerization and membrane insertion during the formation of pores [18,68]. Enterotoxin binds to claudin receptors, forming pores that allow calcium influx, which activates calpain and ultimately leads to cellular apoptosis [18,68,69]. This excessive calcium influx, coupled with IP3 activation, results in cytoplasmic calpain activation, instigating necrosis by releasing mitochondrial cytochrome c and prompting apoptosome formation [69,70,71,72]. This toxin is implicated in food poisoning and diarrhea [18,70]. The novel enterotoxin BEC (also known as CPILE) has emerged as a significant research focus due to its distinctive characteristics and potential role in gastrointestinal pathophysiology [18,51,73]. BEC/CPILE is identified as a heat-stable toxin produced by certain strains of bacteria, particularly in the context of foodborne illnesses and enteric infections [18,51,73]. Its mechanism of action involves the disruption of intestinal epithelial cell integrity, leading to increased permeability and potentially contributing to symptoms such as diarrhea and abdominal cramping [18,51,73]. Research efforts are ongoing to elucidate its precise biochemical pathways and clinical implications, aiming to inform better diagnostic and therapeutic strategies in infectious disease.

Epsilon toxin, produced exclusively by type B and D isolates, is activated by enteric proteases. It causes increased mucosal permeability, resulting in hemorrhagic colitis and edema [18,40,50,74,75]. Epsilon toxin initiates its action by binding to the MAL protein and the P2 receptor (P2R) on the cell surface [18,50]. Subsequently, the activation of neutral sphingomyelinase (NSMase) leads to sphingomyelin (SM) hydrolysis and the formation of ceramide (CER), culminating in epsilon toxin oligomerization, the exposure of phosphatidylserine (PS), and potential cell death [18,40,50,74,75]. The oligomerization of epsilon toxin creates heptameric pores, causing the rapid efflux of K^+^ and the influx of Cl^−^ and Na^+^, followed by increased Ca^2+^ levels [18,40,50,74,75]. Epsilon toxin also induces the translocation of the apoptosis-inducing factor (AIF), a caspase-independent cell death factor, from the mitochondria to the nucleus. This disruption of the intestinal mucosa leads to enterocolitis, characterized by the extravasation of red blood cells and the loss of essential electrolytes [18,40,50,74,75].

Iota toxin, produced exclusively by CP type E strains, consists of two components: an enzyme component (Ia) and a binding component (Ib) [18,47,76,77,78,79,80]. Iota toxin causes the depolymerization of the actin cytoskeleton through adenosine diphosphate (ADP-ribosylation) [50,76,77,78,79,80], thereby altering the cell morphology and disrupting intercellular tight and basolateral junctions [76,77,78,79,80]. This disruption leads to increased paracellular permeability in cultured intestinal cells in vitro. The cell death mechanisms associated with iota toxin in target cells include characteristics of both necrosis and apoptosis

Lambda toxin, produced by CP, is a protein toxin categorized as a β-toxin and is a major virulence factor in CP infections, including GG and other clostridial diseases [16,18,48,75]. Lambda toxin exerts its pathogenic effects by disrupting cell membranes and causing tissue damage [16,18,48,75]. It has a specific affinity for binding to various types of cells, including red and white blood cells, platelets, and endothelial cells lining blood vessels [16,18,48,75].

The mechanism of action of lambda toxin involves initial binding to specific receptors on the surfaces of target cells. This binding triggers the formation of toxin complexes called oligomers, which then integrate into the lipid bilayer of the cell membrane [16,18,48,75]. Once integrated, these oligomers disrupt the cellular integrity, leading to cell lysis (rupture) and subsequent tissue damage [16,18,48,75].

In addition to lambda toxin, CP produces other toxins that contribute to its pathogenicity, including kappa toxins (collagenase and gelatinase). These enzymes are responsible for the destruction of collagen and gelatin, which are important components of blood vessels and connective tissue structures [49]. Meanwhile, Mu, Nu, and Phi toxins are involved in processes such as hemolysis (breaking down red blood cells), tissue necrosis, and the destruction of cellular components [16,18,49]. Together, these toxins play crucial roles in the severe tissue damage and systemic effects observed in infections caused by CP, including GG [49]. Understanding their mechanisms of action is essential in developing effective treatment strategies against these infections.

Delta toxin, produced by certain strains of CP, is a 32 kDa single-chain protein associated with pathogenicity in type B and C strains [18,81,82]. The gene responsible for delta toxin (cpd) is typically located on plasmids, although these plasmids are not fully characterized yet. Delta toxin exerts its effects by forming relatively large pores (~4 nm in diameter) on biological membranes [18,81,82]. Research suggests that ganglioside GM2 may act as a potential cell surface receptor for delta toxin, facilitating its binding and pore formation on host cells [18,81,82]. The formation of these pores disrupts the cellular integrity and function, contributing to the pathogenesis of diseases caused by CP strains that produce delta toxin [18]. In animal models, such as mouse intestinal loops, delta toxin has been shown to induce fluid accumulation and cause damage to the intestinal tissue [18,81,82]. This ability to disrupt the intestinal integrity underscores its role in the pathophysiology of intestinal diseases associated with certain CP infections.

The necrotic enteritis B-like toxin (NetF) is associated with avian necrotic enteritis, particularly affecting chickens. This toxin is a 33 kDa single-chain protein that shares homology with various pore-forming toxins, including 40% identity with CP delta toxin (CP delta toxin) [18,81,83,84]. The structure of NetF consists of four domains: the β-sandwich, latch, rim, and pre-stem domains [83]. NetF functions by forming pores in cell membranes, disrupting cellular communication and potentially leading to cellular apoptosis (programmed cell death) [83]. This disruption of cellular signaling pathways contributes to the pathogenesis of necrotic enteritis in birds. However, it is important to note that NetF is specific to avian species and is not associated with human disease [18]. Therefore, while it poses a significant health concern in poultry, it does not present a risk to human health [18].

Our aim is to provide a comprehensive review of clostridial myonecrosis, encompassing its epidemiology, pathogenesis, clinical presentation, and current treatment strategies, with a particular focus on toxigenomics and the molecular mechanisms underlying toxin production and action. By exploring the genetic and biochemical pathways involved in Clostridium species’ virulence, we aim to elucidate the precise pathophysiology of the disease. This detailed understanding will inform the development of targeted therapeutic interventions and preventive measures. Additionally, we will examine emerging treatment modalities, including antimicrobial agents and hyperbaric oxygen therapy. Ultimately, this review aims to enhance clinical practice and guide future research efforts to mitigate the impact of this fatal disease in humans.

## 2. Types

Understanding the different types of GG and their underlying etiology is crucial for accurate diagnosis and optimal management.

### 2.1. Traumatic Gas Gangrene

Traumatic GG is a severe and rapidly progressing infection primarily caused by CP, accounting for over 80% of cases [1,2,85,86]. Other Clostridium species, like *Clostridium septicum (CS)*, *Clostridium novyi (CN)* type A, and *Clostridium histolyticum*, can also cause these infections, albeit less commonly [87,88]. Infections typically occur following traumatic injuries such as crush injuries, compound fractures, or penetrating wounds, where CP spores are introduced into devitalized tissue, creating an anaerobic environment that promotes bacterial growth and toxin production [1].

The pathogenesis of traumatic GG involves several mechanisms, including toxin-induced microvascular thrombosis [1]. This process reduces tissue perfusion, leading to hypoxia and the subsequent necrosis of affected tissue [1,2,55]. The clinical manifestations include severe pain that is disproportionate to physical findings, rapid tissue necrosis, gas production within tissue (consisting of 5.9% hydrogen, 3.4% carbon dioxide, 74.5% nitrogen, and 16.1% oxygen), and systemic toxicity marked by fever, tachycardia, hypotension, and shock [86,87,88,89]. Treatment involves emergent surgical intervention to debride necrotic tissue and reduce the bacterial load, coupled with high-dose antibiotics targeting anaerobic bacteria like CP [2]. The role of hyperbaric oxygen therapy may be considered to enhance tissue oxygenation and inhibit bacterial growth [89,90,91]. Prompt diagnosis and treatment are crucial to prevent systemic complications such as sepsis, multi-organ failure, and death. A comparison to conditions involving acute arterial thrombosis, where intense pain and tissue necrosis occur due to the occlusion of the blood supply, underscores the critical nature of GG and the need for immediate medical intervention to preserve tissue viability and ensure patient survival.

The clinical presentation of traumatic GG is marked by severe pain disproportionate to physical findings, rapidly progressing tissue necrosis, and the presence of crepitus (gas bubbles) under the skin [1,2,86]. Systemic effects can include fever, tachycardia, hypotension, and shock due to the toxins released by the bacteria. Diagnosis is primarily clinical, supported by imaging such as X-rays or CT scans showing gas within the affected tissue, and laboratory findings such as leukocytosis and metabolic acidosis.

The treatment of traumatic GG is emergent and involves surgical exploration and debridement to remove necrotic tissue and reduce the bacterial load [2]. Antibiotic therapy targeting anaerobic bacteria, such as high-dose penicillin or broad-spectrum antibiotics like carbapenems or clindamycin, is essential. Hyperbaric oxygen therapy may be used adjunctively to enhance tissue oxygenation, inhibit anaerobic bacterial growth, and promote wound healing [91].

The complications of untreated or inadequately treated GG can be severe, including limb loss, septic shock, multi-organ failure, and death [1,92,93,94,95]. The prognosis heavily relies on early diagnosis and the prompt initiation of treatment. Prevention strategies emphasize meticulous wound care, including the thorough cleaning and debridement of traumatic or surgical wounds to minimize bacterial contamination [2]. In high-risk situations, such as contaminated wounds or immunocompromised patients, prophylactic antibiotics may be considered. Ongoing research into vaccines targeting Clostridium species holds promise for future preventive measures against traumatic GG [30,31,56].

### 2.2. Spontaneous or Hematogenous Gas Gangrene

Spontaneous or hematogenous GG differs from traumatic GG in its underlying causes and clinical presentation [1,2,5,18]. It typically arises from the hematogenous spread of bacteria, notably Clostridium species such as CS, from a primary source of infection or colonization. Other Clostridium species, including CP, CN type A, and *Clostridium histolyticum*, may also be involved, albeit less frequently [2].

Common sources include gastrointestinal malignancies, colonic diverticulitis, or other gastrointestinal pathologies [2]. Patients with compromised immune systems, such as those undergoing chemotherapy or with poorly controlled diabetes mellitus, are particularly susceptible to the development of spontaneous GG due to their increased vulnerability to bacterial dissemination [2,96]. Clinically, spontaneous GG may present with a more insidious onset compared to traumatic GG [97]. Patients often exhibit systemic signs such as fever, chills, and malaise, reflecting the hematogenous dissemination of bacteria and systemic toxin effects [97]. Localized symptoms include pain, swelling, and signs of tissue necrosis at the site of infection, developing gradually over time as the infection progresses. The diagnosis of spontaneous GG relies on clinical suspicion, especially in patients with underlying malignancies or immunosuppressive conditions presenting with signs of tissue necrosis and gas formation [2]. Imaging studies such as CT scans can reveal gas within tissue, aiding in diagnosis and in assessing the extent of tissue involvement. Microbiological cultures of blood and the affected tissue confirm the presence of Clostridium species and guide antibiotic therapy.

Treatment involves urgent surgical intervention to debride necrotic tissue and remove the source of infection [6]. Antibiotic therapy targeting anaerobic bacteria, initiated empirically with agents like high-dose penicillin, cephalosporins, or carbapenems, is essential and adjusted based on the culture results [2,97]. Furthermore, clindamycin or linezolid aids in halting toxin production or its effects on the tissue. Supportive care includes aggressive fluid resuscitation and the management of systemic symptoms.

The prognosis depends on early diagnosis and the prompt initiation of treatment. The mortality rates can be high, particularly in immunocompromised patients or those with advanced malignancies. Complications such as sepsis, multi-organ failure, and death emphasize the importance of timely and comprehensive management.

## 3. Epidemiology

In the United States, the incidence of myonecrosis is roughly 1000–3000 cases per year, while the global incidence is approximately 0.4 per 100,000 annually [1,5]. GG has historically been recognized for its significant incidence during wartime, with relatively few civilian cases reported. During World War I, GG complicated approximately 6% of open fractures and 1% of all open wounds, reflecting its frequent occurrence under battlefield conditions [98]. Over subsequent conflicts, including World War II, the Korean War, and the Vietnam War, the incidence steadily decreased to 0.7% and 0.2% and notably declined to 0.002%, respectively [98]. By the Falklands War in 1982, no cases of GG were reported, highlighting advancements in trauma care, antibiotics, and wound management that contributed to reduced infection rates in modern military settings [98].

In a study comparing the survival times after the onset of traumatic and non-traumatic GG, it was found that traumatic GG patients had an average survival time of 15 h, whereas those with non-traumatic gas gangrene had a significantly shorter average survival time of 8 h [1,2,5]. This highlights the critical nature of GG, particularly in non-traumatic cases, which may progress more rapidly, possibly due to the earlier systemic dissemination of bacteria or underlying conditions predisposing patients to infection. In a study involving 1970 earthquake survivors, GG was observed in 0.96% of patients [5]. Another study focusing on 226 patients from the same earthquake highlighted the importance of rapid screening, isolation, surgical debridement, amputation when necessary, and intensive supportive treatment for the successful management and containment of the disease [99].

A study on GG patients exhibited an 80% mortality rate, contrasting that of 0% in necrotizing fasciitis patients, where limb salvage was successful in eight cases, with one amputation [5]. With optimal care, including early detection, surgical intervention, antibiotic therapy, and hyperbaric oxygen treatment, the mortality rate ranges from 20% to 30%, and, in some studies, it is as low as 5% to 10% [5]. If left untreated, the disease is universally fatal. Certain host factors, such as an immunocompromised state, diabetes mellitus, and spontaneous infections, can elevate the mortality rates to 67% or higher [1,2,5]. Infections affecting the abdominal soft tissue or chest wall can result in mortality rates as high as 60%, contrasting those for extremity infections, which have more favorable mortality rates of 5% to 30% [5].

## 4. Clinical Presentation

**Figure 2:**
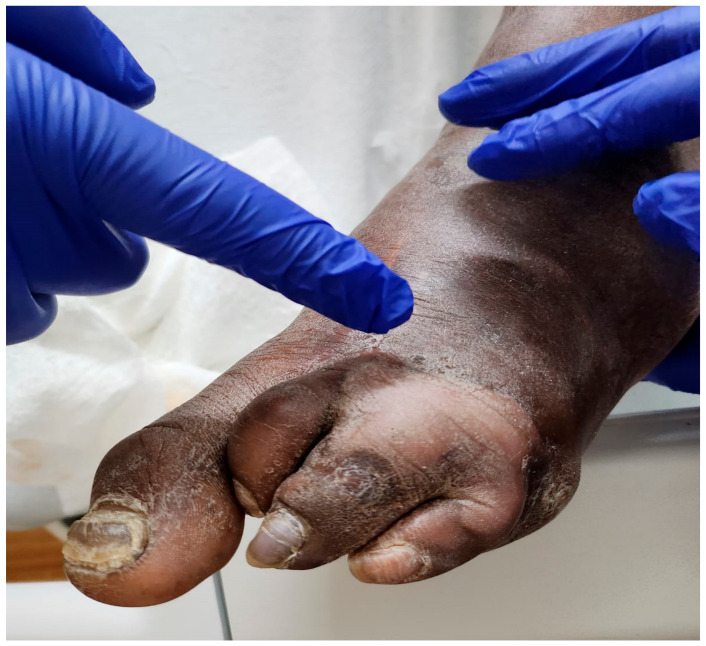
The finger points to the swelling on the lower part of the foot.

The diagnostic criteria for GG involve a combination of the clinical presentation, microbiological findings, imaging studies, and laboratory tests. Regarding the clinical manifestations of the two major types of clostridial myonecrosis, traumatic GG patients typically present with the sudden onset of severe pain localized to the injured site [100,101]. The incubation period for the pathogen itself is typically less than 24 h, contingent upon the degree of vascular compromise and the size of bacterial inoculation [1,102]. Patients commonly present with severe, disproportionate pain that exceeds the physical findings at the injury site. Rapid and progressive swelling, accompanied by tense, shiny, and discolored skin (often bronze or purple), is characteristic due to edema or bullae (Figure 2) and hemorrhage. Palpable crepitus, caused by gas bubbles under the skin resulting from CP or other clostridial species, is a hallmark sign [2]. Foul odors emanating from the infection site, attributed to the release of malodorous gases by bacterial toxins, are characteristic. Systemic signs of sepsis, such as fever, tachycardia, and hypotension, may develop, indicating the severity of the infection.

Spontaneous GG patients typically exhibit pronounced systemic symptoms of sepsis compared to traumatic cases [1,103]. The rapid onset of localized swelling, discoloration, and tenderness at the site of infection are common. Similar to traumatic GG, palpable crepitus may be present due to gas production within the affected tissue [101,102,103]. The dissemination of GG to the bone, resulting in osteomyelitis, is a possibility in non-traumatic cases, as evidenced by the presence of air-filled bullae, as observed in a foot presentation [1,2]. In rare instances, initial presentations may include confusion and other indicators of an altered mental state [2].

For example, one of our patients, a 40-year-old African American male, presented to the emergency department (ED) with fever and palpitations and was diagnosed with sepsis. Management in the ED included antibiotics and fluid resuscitation. The vital signs showed hypotension (100/61 mmHg), a temperature of 38.6 °C, a respiratory rate of 21 breaths per minute, and a pulse rate of 101 beats per minute. The physical examination revealed a toxic appearance, a 3 × 5 cm ulcer on the left plantar area with discharge, skin desquamation, and dark bullae on the posterior surface, with audible crepitus upon superficial examination. The peripheral pulses were preserved, and the remainder of the physical exam, including the neurological assessment, except for a sensation impairment in the affected area, was unremarkable. Four days prior, the patient had presented with left foot pain aggravated by movement, accompanied by shortness of breath and fatigue. Initial investigations showed a positive blood culture for CP, leukocytosis with neutrophil predominance on complete blood count, and X-ray evidence of gas within the left foot tissue. Additionally, elevated blood urea nitrogen, creatinine, and lactic acid levels were noted, while the other blood and urine laboratory results were within normal limits.

GG presents with distinct clinical features that differentiate it from other soft tissue infections. Key considerations in differential diagnosis include necrotizing fasciitis, which can exhibit similar rapid progression and tissue destruction but often lacks the characteristic gas production seen in GG [1,91]. Cellulitis, while also causing localized inflammation, typically lacks the severe pain that is disproportionate to the physical findings and the systemic toxicity often seen in GG [1]. Deep vein thrombosis and compartment syndrome present with localized pain and swelling but do not manifest with extensive tissue gas formation or the rapid progression to tissue necrosis seen in clostridial infections [2]. Additionally, Fournier gangrene, involving the perineal and genital regions, can present similarly with rapid necrosis and systemic signs, necessitating differentiation based on the anatomical location and clinical context [1,2]. Prompt differentiation is crucial for the initiation of appropriate treatment, including early surgical intervention and antibiotic therapy tailored to cover anaerobic bacteria like Clostridium species in GG cases.

## 5. Diagnostic Steps

The diagnosis of GG is based on a combination of clinical evaluation, medical history, and laboratory tests. Given the rapid progression and potential severity of the condition, a prompt and accurate diagnosis is crucial for effective treatment. Below are the key aspects of diagnosing GG.

Medical History: Gathering information about recent trauma, surgery, or other procedures involving the affected area can help to establish a link between potential bacterial exposure and the development of GG. This history can provide insights into the potential entry point for bacteria.

Clinical Presentation: The characteristic clinical features of GG, including severe pain, swelling or bullae (blisters filled with gas; Figure 2), tenderness, crepitus (gas production), a foul odor, and tissue necrosis, are important diagnostic clues (as illustrated above). The rapid onset and progression of these symptoms, often accompanied by systemic signs like fever and tachycardia, raise suspicion for GG [2].

Physical Examination: A thorough physical examination of the affected area is essential. Palpation of the tissue can reveal the presence of crepitus caused by gas accumulation. The appearance of the tissue, including color changes, blisters, and signs of necrosis, also aids in diagnosis [1].

**Figure 3:**
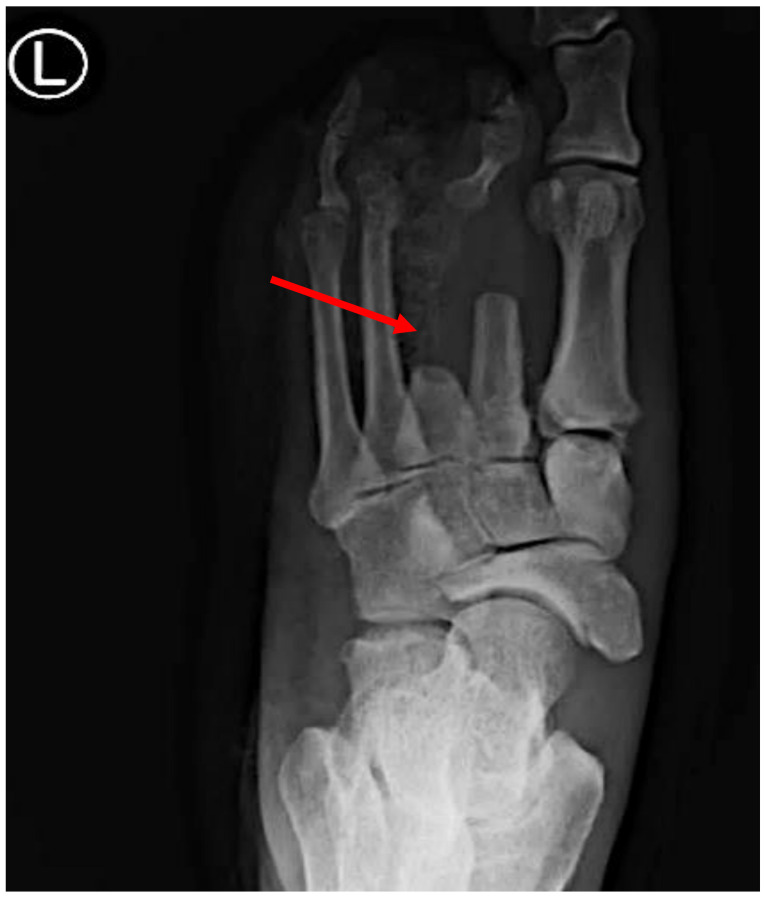
The red arrow points to the presence of gas in a foot with a history of partial bone amputations.

Imaging Studies: Imaging techniques like X-rays, ultrasound, or computed tomography (CT) scans may be used to assess the extent of tissue involvement, gas accumulation, and any associated complications, such as gas in the soft tissue or the involvement of deeper structures (Figure 3). Furthermore, magnetic resonance imaging (MRI) is essential in the management of GG by accurately delineating soft tissue involvement and detecting muscle edema, necrosis, and gas formation early on [1]. It plays a crucial role in differentiating GG from other infections, guiding surgical planning, and assessing the treatment response through the detailed evaluation of the extent and severity of muscle damage. MRI also facilitates targeted biopsy or drainage placement when needed, making it invaluable in both the diagnosis and therapeutic monitoring of this severe soft tissue infection.

Laboratory investigations are pivotal in confirming the diagnosis of GG and informing treatment strategies. These tests encompass a range of modalities.

Blood Tests: Complete blood count (CBC), blood cultures, and blood chemistry analyses serve as valuable tools in assessing systemic inflammation, detecting an infection, and evaluating potential organ involvement.

Tissue Culture: Obtaining samples of infected tissue for bacterial culture and sensitivity testing enables the precise identification of the causative bacteria and facilitates the selection of appropriate antibiotics [1].

Gas Detection: GG is typified by the production of gas within the affected tissue. Radiographic imaging techniques such as plain X-rays may unveil the presence of gas pockets, while ultrasound or CT scans can directly visualize the gas within the tissue, aiding in diagnosis and treatment planning [1,2].

Surgical Exploration: In some cases, the surgical exploration of the affected area may be necessary to confirm the diagnosis and assess the extent of tissue damage. This procedure involves the direct visualization and palpation of the tissue, which can aid in identifying gas, necrosis, and the severity of the infection.

## 6. Etiology and Management

### 6.1. Etiology and Pathogenesis

GG is a highly lethal necrotizing infection of the skeletal muscle and subcutaneous tissue, predominantly caused by CP type A [18]. This condition occurs when type A vegetative cells or spores invade traumatic wounds, leading to the rapid multiplication of vegetative cells and subsequent toxin production [1]. These toxins induce swift, severe, and extensive necrosis in the affected tissue [16,18]. Type A strains of CP are also implicated in various gastroenteric syndromes in animals, although their exact role in these diseases remains controversial [18]. One of the main challenges in attributing the disease to type A isolates is their ubiquitous presence in the environment and in the intestines of many animal species, rendering their isolation from gastrointestinal samples diagnostically insignificant. However, an exception may be type A isolates encoding the recently discovered NetF toxin [16,18,83]. These strains are suggested to be associated with canine hemorrhagic gastroenteritis and necrotizing enterocolitis in foals, with the link being primarily epidemiological. These NetF toxin-producing strains appear to be more prevalent in animals afflicted by these specific diseases [83].

Infections by CP type B strains primarily affect sheep, where the disease is known as lamb dysentery [13,18]. This condition is characterized by necro-hemorrhagic enteritis and, in rare cases, focal symmetrical necrosis, thought to be caused by epsilon and beta toxins, respectively [18]. Recently, CP type B was found in the feces of a human patient with multiple sclerosis. Additionally, epsilon toxin serum antibodies have been detected in patients with multiple sclerosis [16,18]. These findings have led to speculation that epsilon toxin may be associated with the pathogenesis of multiple sclerosis, although definitive evidence supporting this hypothesis is still lacking [18].

Infections caused by CP type C strains result in necrotizing enteritis and enterotoxemia across various mammalian species, including humans, with a particular predilection for neonates [18,104,105,106,107,108]. This predisposition is linked to beta toxin’s sensitivity to trypsin, a natural defense mechanism against the disease [18,104,105,106,107,108]. Neonates, especially those ingesting colostrum, are more vulnerable because colostrum acts as a potent trypsin inhibitor, allowing beta toxin to exert its effects more readily [18]. In humans, foodborne type C disease was historically common among malnourished individuals in post-World War II Germany, where it was referred to as Darmbrand. This condition, known medically as enteritis necroticans or PigBel, exhibited high prevalence in Papua New Guinea during the 1960s [18,104,105,106,107,108]. Although it is no longer endemic, sporadic cases continue to occur in this region [18]. PigBel primarily affected malnourished children, who likely had low levels of trypsin due to poor diets and the consumption of sweet potatoes, which contain a potent trypsin inhibitor [18]. These children developed the disease after ingesting incompletely cooked meat, often pork, contaminated with type C strains [16,104]. Rare cases of type C infections have also been documented in individuals with diabetes or other pancreatic diseases, indicating a broader potential risk beyond malnutrition and dietary factors [18].

CP type D strains lead to enterotoxemia in animals, with no reports indicating that they can cause human infection [13,16,18,105]. The role of CP type E in human and animal diseases remains not fully understood [16,18]. Although there have been a few documented cases of type-E-associated disease in various animal species, the majority of these diagnoses were based on the isolation of the microorganism from the intestinal contents of sick animals [18]. Since CP type E is commonly found as a normal inhabitant in the intestines of healthy individuals across many animal species, its mere isolation does not fulfil the diagnostic criteria for type E disease [18]. Thus, additional diagnostic markers and evidence are required to conclusively attribute the disease to type E strains [18].

CP type F strains are major gastrointestinal pathogens, primarily due to the strains’ production of highly resistant spores that survive in improperly stored or undercooked foods and their rapid bacterial growth in these environments [13,16,18]. Outbreaks often occur in institutions where large amounts of food are prepared and held for extended periods.

Type F food poisoning begins with the ingestion of contaminated food, leading to diarrhea and abdominal cramps within 12–24 h, usually resolving in a day [13,16,18]. However, fatalities can occur in the elderly or those with preexisting conditions. In psychiatric facilities, fatalities have been linked to patients with constipation, where the toxin is absorbed into the bloodstream, affecting organs like the liver and kidneys [18,109,110]. Type F strains also cause 5–15% of non-foodborne gastrointestinal diseases, including antibiotic-associated diarrhea and sporadic diarrhea [18,111]. These strains typically carry a plasmid-borne *cpe* gene and produce less resistant spores compared to those causing foodborne illness [18,112,113].

CP type G strains cause necrotic enteritis (NE), a significant disease affecting poultry worldwide and resulting in estimated annual economic losses of USD 5 billion [18,109]. NE can manifest as subclinical, primarily impacting weight gain, or clinical, with symptoms including reluctance to move, diarrhea, decreased appetite, huddling, dehydration, or sudden death without prior symptoms [109]. Acute NE lesions are found mainly in the jejunum and ileum, characterized by gas-distended intestines filled with dark brown, semi-liquid material and an ulcerated mucosa covered by a fibrino-necrotizing membrane [18,109]. Hemorrhage is uncommon. In subacute and chronic cases, similar lesions occur with thickened intestinal walls and multiple mucosal ulcerations [109]. Some affected chickens also exhibit cholangiohepatitis, presenting as enlarged, firm, pale livers with scattered yellow necrotic foci [109].

Etiologically, CS infections typically arise from its ability to enter the bloodstream through breaches in the intestinal mucosa, often caused by trauma, surgery, or underlying gastrointestinal malignancies [1,18,114]. Unlike other clostridial infections, which primarily affect traumatized tissue, CS can cause spontaneous infections in immunocompromised individuals or those with underlying conditions such as colorectal cancer, leukemia, or diabetes mellitus [114]. This bacterium produces several toxins and enzymes that contribute to tissue necrosis and systemic toxicity, including alpha toxin, collagenase, and hyaluronidase [18]. In cases of GG, the presence of gas in the tissue is a diagnostic step and is due to the bacterium’s ability to ferment carbohydrates and produce gases like hydrogen and carbon dioxide [18]. Septicemia and toxemia are common complications, leading to shock and multi-organ failure if not promptly treated with aggressive surgical debridement and antibiotic therapy [114].

CS-associated aortitis represents a rare yet highly lethal condition that requires urgent interdisciplinary evaluation and intervention to potentially achieve long-term survival outcomes [1]. It is rarely found to have concomitant aortitis and GG [100]. Early diagnosis is crucial, often involving advanced imaging techniques such as CT scans or MRI to assess the extent of aortic involvement and guide treatment decisions [100]. The prompt initiation of broad-spectrum antibiotics that are effective against anaerobic bacteria like Clostridium species, along with surgical interventions such as debridement and possibly aortic repair or reconstruction, forms the cornerstone of treatment [1].

CN is associated with several veterinary diseases, particularly in sheep and cattle [8]. It produces potent toxins, including alpha and beta toxins, which contribute to its pathogenicity [8,18]. Infections typically occur following the ingestion of spores from the environment, leading to localized tissue necrosis and potentially fatal toxemia [8]. CN is also recognized for its role in causing hepatic necrosis in animals, often associated with liver fluke infestations [18]. In humans, CN infections are rare but can occur in immunocompromised individuals or those with underlying liver disease [18].

*Clostridium histolyticum* is capable of producing several potent enzymes and toxins that contribute to tissue destruction and disease pathogenesis [115]. It is most notably associated with causing GG in humans, a severe condition characterized by rapid tissue necrosis and gas formation within the affected tissue [18,115]. The bacterium produces collagenase and other tissue-destructive enzymes that aid in breaking down connective tissue and facilitating the spread of the infection [115].

### 6.2. Treatment

The management of GG typically involves a combination of medical (antibiotics and hyperbaric oxygen) and surgical interventions aimed at controlling the infection and preventing systemic complications [1]. Antibiotic choices typically include cephalosporins like ceftriaxone 1 g IV every 12 h for 10–14 days or penicillin G IV 4 g every 4 h for 10–14 days; others include broad-spectrum options such as Zosyn (piperacillin-tazobactam) 4.5 g IV every 6 h for 10–14 days [116,117]. These are often supplemented with clindamycin 900 mg IV every 8 h for 10 days or linezolid 600 mg IV every 12 h for 10 days to inhibit toxin production [1,116,117]. Metronidazole 500 mg IV every 8 h for 10–14 days is frequently added for its anaerobic coverage [116,117]. Sometimes, intravenous vancomycin 1 g every 12 h is used as an initial treatment option in combination with clindamycin and metronidazole [116,117].

Many clinicians prefer a regimen combining penicillin, clindamycin, and metronidazole due to their efficacy against clostridial species and ability to neutralize toxins, tailored to the severity of the infection and patient-specific factors. However, carbapenem (meropenem 1 g Q8H IV) can be started initially, especially in severely immunocompromised patients, in combination with clindamycin and vancomycin [116,117]. Other antibiotics, such as cefepime, fluoroquinolones, cefiderocol, cefoxitin, cefotetan, and beta-lactam/beta-lactamase inhibitor combinations, provide broader Gram-negative coverage compared to Gram-positive coverage [116,117,118].

While less commonly used, antitoxin therapy can be effective in specific situations. Antitoxins are antibodies designed to neutralize particular toxins. For instance, alpha antitoxin can counteract the effects of CPA, and epsilon toxin can be neutralized by antitoxin IgY [119]. This therapy is most effective when given early in the infection, before extensive tissue damage occurs. However, the availability of specific antitoxins is limited, and their use is typically reserved for severe or refractory cases.

Hyperbaric oxygen therapy (HBOT) has been involved in various wound healing and gastrointestinal disorders, as well as necrotizing soft tissue infections, over the last few decades [89,90,91]. HBOT’s role in the treatment of GG occurs through several mechanisms. By exposing patients to 100% oxygen at increased atmospheric pressure (typically two to three times higher than normal), HBOT enhances oxygen delivery to hypoxic tissue [89,90,91]. This elevated oxygen tension inhibits the growth and proliferation of anaerobic bacteria like Clostridium species, which thrive in low-oxygen environments [89,90,91]. Moreover, HBOT promotes the formation of reactive oxygen species (ROS) within tissue [89,90,91]. These ROS are toxic to bacteria and aid in their destruction by disrupting cellular membranes and proteins. Additionally, HBOT assists in wound healing by stimulating angiogenesis, enhancing fibroblast activity, and reducing tissue edema and inflammation [89,90,91]. Clinically, HBOT is used adjunctively with surgical debridement and antibiotic therapy to improve outcomes in severe cases of GG [89,90,91]. It helps to preserve viable tissue, mitigate the spread of infection, and reduce the need for limb amputation [89,90,91]. The combined effects of increased tissue oxygenation, the bactericidal action of ROS, and supportive tissue repair mechanisms underscore the scientific rationale behind hyperbaric oxygen therapy in the management of GG [89,90,91].

The surgical management of GG involves several critical steps aimed at removing necrotic tissue, controlling the infection, and preserving as much viable tissue as possible to optimize patient outcomes [120]. Initially, thorough surgical debridement is paramount, involving the excision of all visibly necrotic and contaminated tissue [1,120]. This procedure not only removes the anaerobic environment necessary for bacterial growth but also reduces toxin production and prevents the further spread of the infection [1,5,120].

Following debridement, the meticulous irrigation of the wound with copious amounts of sterile saline or antiseptic solutions helps to flush out the remaining bacteria and debris [120]. This step is crucial in reducing the bacterial load and minimizing the risk of recurrent infection. Some surgeons may choose the use of wound vacuums or negative pressure wound therapy to promote wound healing and reduce the risk of secondary infection. In cases where extensive tissue damage or compartment syndrome is present, fasciotomy—a surgical procedure used to relieve pressure within muscle compartments—may be necessary to improve tissue perfusion and prevent further necrosis. This can be essential in preventing the progression of gangrene and preserving limb function [1].

Throughout the surgical procedure, careful attention is paid to achieving hemostasis to minimize bleeding and optimize wound healing. Depending on the extent of tissue involvement and the patient’s condition, reconstructive surgery or skin grafting may be considered at a later stage to restore form and function to the affected areas. Overall, the surgical management of GG requires a comprehensive approach, often involving collaboration between surgical teams, infectious disease specialists, and critical care providers. Early intervention, thorough debridement, effective irrigation, and appropriate wound care are essential in reducing the morbidity and mortality associated with this potentially life-threatening condition.

The development of vaccines against other Clostridium toxins, such as those associated with GG or other clostridial infections, is an area of ongoing research [30,31]. These vaccines aim to mitigate the effects of toxins that cause tissue necrosis and systemic toxicity.

Rehabilitation following GG focuses on comprehensive recovery strategies tailored to the individual’s needs [121]. Central to this process is physical therapy, which aims to restore strength, flexibility, and mobility in the affected limbs through targeted exercises and gradual progression. Wound care is also pivotal, involving meticulous dressing changes and monitoring to promote healing and prevent complications [121]. Psychological support plays a crucial role in addressing the potential anxiety or trauma associated with the illness and its treatment. Additionally, functional rehabilitation may include learning adaptive techniques or using assistive devices to regain independence in daily activities [120]. Nutritional counseling ensures adequate dietary intake to support tissue repair and overall recovery. Regular follow-up with healthcare providers ensures the ongoing monitoring and management of any lingering issues or complications, ultimately facilitating optimal recovery and improving quality of life following gas gangrene.

Patients with GG require close monitoring in the intensive care unit to assess the progress of treatment, manage any complications, and adjust the therapy as needed. Routine evaluations are crucial to ensure that the infection is managed and the overall health of the patient is improving. With advancements in technology, including improved diagnostic tools and therapeutic strategies, along with a deeper understanding of its pathophysiology and treatment modalities, the mortality and morbidity associated with GG have seen a significant decline over the past few decades. These developments have enabled earlier diagnosis, more effective surgical interventions, and the use of targeted antibiotic therapies, leading to better outcomes and improved quality of care for affected individuals. However, delayed treatment can lead to rapid deterioration and severe complications. Due to the complexity of GG treatment, a team of healthcare professionals, including surgeons, infectious disease specialists, anesthesiologists, and critical care providers, often collaborate to provide comprehensive care.

The emergence of antibiotic resistance in Clostridium species poses a significant challenge in the treatment of gas gangrene. Resistance to commonly used antibiotics, such as penicillin and clindamycin, has been increasingly reported. Various CP resistance genes have been reported in recent years [122,123]. For example, the erm(T) gene was identified in three resistant strains of CP (MLG 1108, MLG 3111, MLG 7009) to erythromycin, while the ant(6)-Ib resistance aminoglycoside gene was detected in CP MLG 2314 [122,123]. This resistance complicates therapeutic strategies and underscores the need for ongoing surveillance and the development of alternative treatment options [122,123]. Research efforts are focused on understanding the genetic and biochemical mechanisms underlying this resistance, including the identification of resistance genes and the role of biofilms in protecting bacteria from antibiotic action.

Given the critical role of toxins in the pathogenesis of GG, developing novel toxin-neutralizing agents represents a promising therapeutic approach. Current research is exploring various strategies to neutralize these toxins and mitigate their effects. These include monoclonal antibodies (mAbs) that specifically target and neutralize Clostridium toxins, small molecule inhibitors that block toxin activity, and synthetic peptides that mimic toxin-binding sites on host cells to act as decoys [122,124,125]. Furthermore, phage therapy is being explored as a means to specifically target and kill Clostridium species. Bacteriophages, or viruses that infect bacteria, could potentially reduce the bacterial load and toxin production without the use of traditional antibiotics [125]. Immunomodulatory agents that modulate the host immune response to reduce inflammation and enhance toxin clearance are also under investigation. These agents aim to bolster the body’s natural defenses against the infection.

CP case studies have highlighted various treatment approaches tailored to manage this life-threatening condition effectively. In one case, a 64-year-old male presented with severe pain, swelling, and discoloration in his left flank, indicative of Clostridium myonecrosis. Following prompt diagnosis through clinical signs and microbiological testing, the patient received high-dose meropenem, clindamycin, and metronidazole. Surgical debridement was necessary to remove the necrotic tissue, and the patient showed a significant improvement post-surgery. This approach emphasizes the critical role of early, aggressive antibiotic therapy combined with surgical intervention [126]. Another approach involved a 32-year-old female who developed Clostridium myonecrosis in the right hand. Treatment included broad-spectrum antibiotics (teicoplanin and meropenem), surgical debridement, and HBOT. Daily sessions of HBOT significantly controlled the infection, preventing the need for amputation and reducing bacterial proliferation and toxin production [127]. In a different case, a 56-year-old female with a history of breast cancer presented with severe right hand pain, discoloration, and swelling; she was diagnosed with Clostridium myonecrosis. After surgical intervention (amputation) and antibiotics (penicillin and clindamycin), the patient survived [128].

Given the severity of Clostridium myonecrosis, the exploration of future prospective therapies holds promise in halting the disease’s progression and reducing the mortality and morbidity rates. For example, stem cell transplantation following surgical debridement may emerge as a viable option to facilitate tissue regeneration and restore lost tissue integrity. Additionally, investigating the potential of radiation therapy using proton machines before surgery could lead to a novel approach to enhance patient outcomes by targeting localized infection sites more precisely and minimizing the collateral damage to healthy tissue. These innovative avenues highlight the ongoing pursuit of advanced treatment modalities aimed at improving the clinical outcomes in the management of this challenging and potentially life-threatening condition.

## 7. Conclusions

GG is a severe and life-threatening bacterial infection primarily caused by CP, a bacterium renowned for its ability to produce a variety of toxins and enzymes that rapidly degrade tissue, leading to necrosis and systemic complications. This infection typically occurs in wounds with a compromised blood supply, creating an anaerobic environment favorable for bacterial growth. Clinical presentation includes severe pain, swelling, tenderness, a foul odor, skin discoloration, and the distinctive crepitus sensation due to gas buildup within tissue. Immediate medical intervention is crucial as GG constitutes a medical emergency. Treatment involves a multifaceted approach combining surgical intervention, antibiotic therapy, and supportive care. Surgical management includes the prompt and extensive debridement of necrotic tissue to remove the bacterial reservoir and toxins, thereby halting disease progression. In severe cases, fasciotomy may be necessary to relieve pressure and restore tissue perfusion. Antibiotic therapy targets CP with broad-spectrum agents such as penicillin, cephalosporins, or carbapenems, often in combination with clindamycin to counteract toxin production. Advancements in medical knowledge and techniques, including hyperbaric oxygen therapy, have improved the outcomes for patients with GG. Hyperbaric oxygen therapy enhances tissue oxygenation, inhibits bacterial growth, and promotes wound healing, particularly in cases where the surgical options are limited. Research continues into novel treatment modalities, including immunotherapy and targeted antimicrobial agents, to address antibiotic resistance and improve patient outcomes. Early diagnosis through clinical suspicion and imaging studies remains critical. The immediate initiation of appropriate treatment significantly reduces the morbidity and mortality associated with GG. Future strategies aim to optimize the therapeutic approaches, enhance the early detection methods, and develop vaccines targeting CP toxins, underscoring ongoing efforts to improve the outcomes in this challenging condition.

## Data Availability

The data presented in this study are available on request from the corresponding authors.
